# MD2 Is a Potential Biomarker Associated with Immune Cell Infiltration in Gliomas

**DOI:** 10.3389/fonc.2022.854598

**Published:** 2022-03-17

**Authors:** Mengya Zhao, Xiaodong Li, Yijun Chen, Shuzhen Wang

**Affiliations:** State Key Laboratory of Natural Medicines and Laboratory of Chemical Biology, School of Life Science and Technology, China Pharmaceutical University, Nanjing, China

**Keywords:** MD2, DNA methylation, glioma, biomarker, macrophage, neutrophil

## Abstract

**Background:**

Glioma is the most common primary malignant tumor in the central nervous system. Myeloid differentiation protein 2 (MD2) acts as a coreceptor of toll-like receptor 4 (TLR4) to mediate innate immune response. However, the actual roles of MD2 in the regulation of progression and immune cell infiltration in gliomas remain largely unclear. This study aims to explore whether MD2 could be an independent prognostic factor through the mediation of immune cell infiltration in gliomas.

**Methods:**

The mRNA expression and DNA methylation differential analyses of MD2 were performed using CGGA, TCGA and Rembrandt databases and survival analyses were performed using Kaplan-Meier plotter. Univariate and multivariate Cox regression was applied to analyze the prognostic value of MD2 and nomograms were constructed to evaluate the clinical value of MD2. Then, Gene Ontology (GO) and Kyoto Encyclopedia of Genes and Genomes (KEGG) were utilized to analyze MD2-related signal pathways. Furthermore, correlations between MD2 and immune cell infiltration were calculated by TIMER and CIBERSOPT. The correlation between MD2 expression and the infiltrations of macrophages and neutrophils was experimentally verified by the knockdown of MD2 expression using small interfering RNA (siRNA) in glioma cells.

**Results:**

We found that MD2 was overexpressed and associated with a poor prognosis in gliomas. Meanwhile, higher expression of MD2 could be a result of lower DNA methylation of *MD2* gene in gliomas. In addition, univariate and multivariate Cox regression analysis indicated that MD2 could be an independent prognostic factor for gliomas. Further functional enrichment analysis revealed that the functions of MD2 were closely related to immune responses. Moreover, the expression level of MD2 was strongly correlated with the infiltration and polarization of pro-tumor phenotype of tumor-associated macrophages and tumor-associated neutrophils in gliomas.

**Conclusions:**

These findings have provided strong evidence that MD2 could be served as a valuable immune-related biomarker to diagnose and predict the progression of gliomas.

## Introduction

Gliomas are the most common primary intracranial tumor in the brain parenchyma, which originates from glial or precursor cells (1–3). According to the 2016 World Health Organization Classification of Tumors of the Central Nervous System classification criteria, gliomas are classified into low-grade glioma (LGG, WHO I-II) and high-grade glioma (HGG, WHO III-IV), and glioblastoma (GBM) is among WHO grade IV (4). Currently, the first-line treatment for gliomas is surgical resection and the combination with radiotherapy or chemotherapy with temozolomide (TMZ), which could improve the patients’ quality of life and lifespans (5–7). However, the majority of glioma patients develop inherent or acquired resistance to TMZ alone or combination therapy, leading to inevitable relapse or malignant progression eventually (8–10). In recent years, immunotherapy with immune checkpoint inhibitors has become an appealing innovative treatment to deal with the tolerance or relapse of glioma by traditional therapies (11, 12). Despite the fact that the FDA-approved nivolumab targeting programmed cell death protein 1 (PD-1) and Ipilimumab targeting CTLA-4 have displayed clinical therapeutic superiority compared to conventional therapies in multiple types of cancers (13–16), the combination of nivolumab and Ipilimumab shows no significant effects on recurrent glioblastoma (17, 18). In gliomas, the status and extent of tumor-infiltrating lymphocytes, such as tumor-associated macrophages (TAMs) and tumor-associated neutrophils (TANs), are highly associated with glioma grade, immune evasion and therapeutic resistances, due mainly to their switch capacity of selectively polarizing between pro-inflammatory subtype (M1 or N1) and immunosuppressive subtype (M2 or N2) under different activation conditions (19–24). In addition, the infiltration amounts of tumor-infiltrating immune cells in gliomas with isocitrate dehydrogenase (IDH) mutation and chromosome 1p/19q codeletion are lower than those with wild-type IDH or 1p/19q non-codeletion (25–27). Regardless of prior association of gliomas with immune cell infiltrations, there are very limited biomarkers that could actually reflect the extent of immune cell infiltration in the tumor microenvironment. Therefore, new immune-related biomarkers are important to the diagnosis and treatment for glioma patients, especially given the lack of reliable and practical biomarkers for gliomas.

Myeloid differentiation protein 2 (MD2) is a secreted glycoprotein, which acts as a coreceptor of toll-like receptor (TLR4) to mediate innate immune and inflammatory responses to bacterial lipopolysaccharide (LPS) (28–30), in which inflammation is suggested to be the major cause for tumorigenesis (31, 32). While previous studies have revealed that TLR4 expression is significantly associated with tumor progression, including colorectal cancer (33), head and neck cancer (34) and ovarian cancer (35). TLR4 has also been reported to be associated with stem cell maintenance and chemoresistance induced by TMZ in gliomas (36, 37). However, as a coreceptor of TLR4, the expression of MD2 in gliomas and the roles played in tumor immunity remain largely unknown. On the other hand, based on the nature of a coreceptor of TLR4 (27–29) and the relationship with drug-resistance (35, 36), we speculated that MD2 could be a potential biomarker for the diagnosis and treatment of gliomas.

In this study, after comprehensively analyzing the levels of mRNA expression and DNA methylation of MD2 in glioma tissues based on CGGA, TCGA and Rembrandt databases, we found that MD2 was significantly overexpressed in gliomas, which was negatively correlated with DNA methylation of *MD2* gene. In addition, the levels of DNA methylation and mRNA expression of MD2 were closely related to clinical malignancy features of gliomas, indicating that MD2 exhibits important prognostic values for gliomas. Further functional enrichment analysis revealed that the functions of MD2 were associated with immune responses. Moreover, the expression level of MD2 was positively correlated with the infiltration of M2-type TAMs and N2-type TANs, whereas its DNA methylation displayed an opposite trend. Intriguingly, reduction of the expression level of MD2 by siRNA resulted in a significant decrease in the secreted factors to influence the infiltration of M2-type TAMs and N2-type TANs by glioma cells, verifying the definitive relationship between MD2 expression and immune cell infiltration.

## Materials and Methods

### Data Collection and Integration

The RNA-seq data and corresponding clinical information were downloaded from CGGA (www.cgga.org.cn) (38), TCGA (http://xena.ucsc.edu/) and Rembrandt (https://wiki.cancerimagingarchive.net/display/Public/REMBRANDT) database, including 713 samples from CGGA database (20 normal samples and 693 glioma samples), 1680 samples from TCGA database (895 normal samples and 685 glioma samples) and 364 samples from Rembrandt database (21 normal samples and 343 glioma samples). Clinical information of the glioma patients consisted of WHO grade, IDH1 status, 1p/19q status, age, gender and overall survival. Some samples with unavailable or unclear clinical information were removed. In addition, the DNA methylation data was also downloaded from TCGA database described above.

### Analysis of Survival Data

All glioma samples were divided into high and low MD2 expression (high methylation or low methylation) groups by the median expression level of MD2 in each database. The association between MD2 expression level (or methylation level) and overall survival in glioma samples were assessed by Kaplan-Meier survival analysis with log-rank test.

### Univariate and Multivariate Cox Regression Analysis

Univariate and multivariate Cox regression was used to examine whether MD2 expression, age, pathological grade, 1p/19q status and IDH mutation were independent prognostic factors in glioma patients based on CGGA and TCGA database. Hazard ratios (HR) and 95% confidence intervals (CI) were calculated in this study.

### Construction of Nomogram

The nomogram was used to predict cancer prognosis individually by incorporating clinical characteristics and risk scores of the patients. The calibration curves were utilized to visualize the deviation of predicted probabilities from what actually happened. The concordance index (C-index) was applied to measure the predictive accuracy of the nomogram. Time-dependent Receiver operating characteristic (ROC) curve was generated using R package survival ROC.

### Analysis of Immune Cell Infiltration

Tumor Immune Estimation Resource (TIMER 2.0) database (http://timer.cistrome.org/) was used to analyze the correlation between MD2 expression and the infiltration of six types of immune cells (B cells, CD4^+^ T cells, CD8^+^ T cells, neutrophils, macrophages and dendritic cells) in LGG and GBM (39). In addition, CIBERSORT (http://cibersort.stanford.edu) was also applied to analyze the relationship between MD2 expression and 22 types of human immune cell subpopulations based on CGGA and TCGA datasets (40). The correlation between MD2 methylation and immune cell infiltration was analyzed by R package EpiDISH at cg13213009 and cg23732024 CpG sites. Estimation of stromal and immune cells in malignant tumor tissues using expression data (ESTIMATE) (https://bioinformatics.mdanderson.org/estimate) was employed to calculate the degree of immune cell infiltration.

### MD2-Related Function Enrichment Analysis

KEGG and GO were applied to assess MD2 associated potential functions in gliomas based on TCGA database with R package ClusterProfiler (41). The correlation between MD2 expression level and immunomodulators was evaluated by TISIDB (http://cis.hku.hk/TISIDB) database in LGG and GBM respectively (42). The 50 interacting proteins with MD2 were collected from STRING (STRING: string-db.org) and top-100 MD2-related genes were obtained from GEPIA2 (gepia2.cancer-pku.cn) (43). The protein-protein interaction (PPI) network was visualized by Cytoscape software (44). Pearson’s correlation analysis was conducted between MD2 and the coincide of interaction and related genes using the GEPIA2 in LGG and GBM respectively.

### Cell Cultures and MD2 Silencing

The glioma cell lines U87 and A172 were purchased from the Chinese Academy of Sciences Cell Bank (Shanghai, China) and cultured in Dulbecco’s Modified Eagle Medium (DMEM) containing 10% fetal bovine serum (FBS), penicillin (100 U/mL) and streptomycin (100 μg/mL) and cultured and humidified incubator which maintained at 5% CO_2_ and 37°C.

The glioma cell lines were transfected with 100 nM siRNA targeting MD2 (sense, 5’-GAAUCUUCCAAAGCGCAAATT-3’) or a non-coding scramble negative control siRNA (sense, 5’- TTCTCCGAACGTGTCACGTTT-3’) using 3 μL RNAi MAX reagent (Invitrogen, USA) in the opti-MEM medium. After 6 h incubation, the media was changed to normal DMEM and then cultured for 48 h.

### Western Blot

Cells were lysed in NP-40 lysis buffer (150 mM NaCl, 100 mM NaF, 50 mM Tris-HCl (pH 7.6), and 0.5% NP-40) with protease inhibitor PMSF (1:100) at 4°C for 15 min. The protein concentration was measured using the BCA protein assay kit (Beyotime, Shanghai, China). The protein samples were subjected to 12% SDS-PAGE and then transferred to PVDF membrane and blocked in TBST with 5% no-fat milk at room temperature for 2 h. The membranes were incubated with primary antibodies at 4°C overnight. Then, the membranes were washed in TBST buffer and incubated with species-match HRP-linked secondary antibody at room temperature for 2 h. Afterwards, membranes were washed three times in TBST buffer, developed using the enhanced chemiluminescence (ECL) reagent (Fdbio science, Hangzhou, China), and captured by Tanon-5200Multi Imaging System (Tanon, Shanghai, China). Antibodies used in this study were as follows: rabbit polyclonal anti-MD2 (1178-1-AP; Proteintech, Chicago, USA), mouse monoclonal anti-β-Tubulin (2128; Cell Signaling Technology, Boston, USA), anti-mouse IgG, HRP-linked antibody (7076S; Cell Signaling Technology, Boston, USA) and anti-rabbit IgG, HRP-linked antibody (7074S; Cell Signaling Technology, Boston, USA).

### Quantitative Reverse-Transcription Polymerase Chain Reaction

Total RNA was extracted from the cells using the TRIzol reagent (Accurate Biology Co. Ltd, Hunan, China) according to the manufacturer’s instruction. Then, cDNA was synthesized by using reverse transcriptase (Vazyme, Nanjing, China). qRT-PCR was carried out using SYBR green supermix (Vazyme, Nanjing, China) with gene-specific primers. β-Actin was used as an internal standard for normalization. The sequences of primers for qRT-PCR are provided in the **Table 1**. Each assay was performed in triplicate and the data were analyzed with the 2^-△△Ct^.

**Table 1:** The primer sequences of indicated genes for qRT-PCR detection.

Gene	Sequences of primers (5’-3’)
MD2-F	AGCTCTGAAGGGAGAGACTGT
MD-R	AGAGCATTTCTTCTGGGCTCC
TLR4-F	TGCGTGAGACCAGAAAGC
TLR4-R	TTAAAGCTCAGGTCCAGGTTC
CSF-1F	CGCCCACTCCGCAGC
CSF-1R	CCAGCCATGTCGTGGGAG
CCL-2F	TCTGTGCCTGCTGCTCATAG
CCL-2R	GGGCATTGATTGCATCTGGC
IL10-F	CGCATGTGAACTCCCTGG
IL10-R	TAGATGCCTTTCTCTTGGAGC
TGF-β-F	GTGGTATACTGAGACACCTTGG
TGF-β-F	CCTTAGTTTGGACAGGATCTGG
CXCL-2F	AACCGAAGTCATAGCCACAC
CXCL-2R	CTTCTGGTCAGTTGGATTTGC
CXCL-5F	TCTGCAAGTGTTCGCCATAG
CXCL-5R	CAGTTTTCCTTGTTTCCACCG
G-CSF-F	TTCCTGCTCAAGTGCTTAGAG
G-CSF-R	AGCTTGTAGGTGGCACAC
GM-CSF-F	CTGAACCTGAGTAGAGACACTG
GM-CSF-R	GCCCTTGAGCTTGGTGAG

### Statistical Analysis

The R software (Version 4.1.0), Graphad Prism 8 software (Version 8.0.2) and Adobe Illustrator software (Version 24.0.2) were used to perform statistical analysis and generate figures. Difference analysis between two groups was analyzed using Student’s *t*-test, and *p* < 0.05 was considered statistically significant. Continuous variable fitting a normal distribution was described as the mean with standard deviation.

## Results

### Differential Expression of MD2 in Glioma Patients

**Figure 1:**
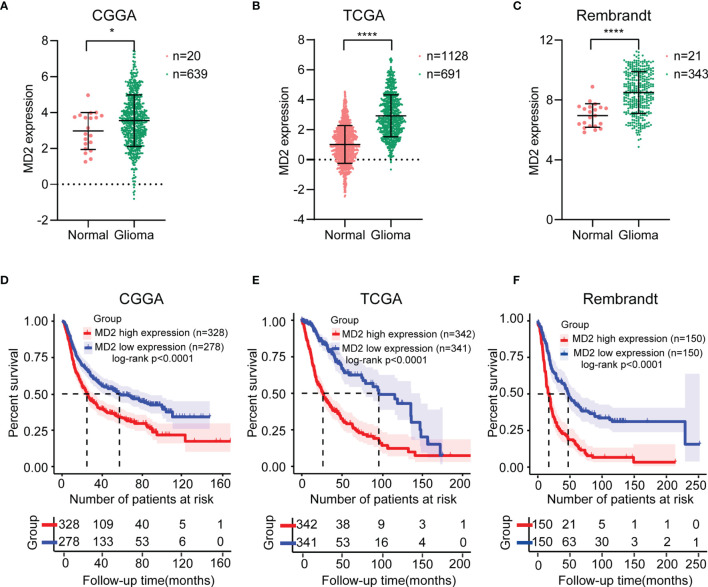
The MD2 expression and survival analysis in gliomas. The mRNA expression differential analysis of MD2 in glioma patients in CGGA **(A)**, TCGA **(B)** and Rembrandt **(C)** databases. Kaplan-Meier survival plots of the associations of MD2 expression with glioma patient overall survival in CGGA **(D)**, TCGA **(E)** and Rembrandt **(F)** databases. * and **** indicate *p* < 0.05 and *p* < 0.0001, respectively.

The expression level of MD2 in glioma tissues and normal brain tissues was analyzed using the data from CGGA databases. The analysis indicated that MD2 was significantly overexpressed in glioma patients in comparison to normal brain tissues (**Figure 1A**). Further analyses of other two databases of TCGA and Rembrandt obtained similar results (**Figures 1B, C**). Next, to analyze the association between the expression level of MD2 and overall survival of glioma patients, Kaplan-Meier plotter analysis was performed with the datasets from CGGA, TCGA and Rembrandt. According to the median expression of MD2 in each dataset, the expression level of MD2 in glioma patients was separated into two groups with high and low expression. The Kaplan-Meier curves showed that high expression level of MD2 was remarkably related to the poor overall survival of glioma patients in CGGA (**Figure 1D**, *p* < 0.0001), TCGA (**Figure 1E**, *p* < 0.0001) and Rembrandt (**Figure 1E**, *p* < 0.0001), respectively. These results indicated that MD2 could function as an oncogene, and its high expression may portend a worse prognosis in gliomas.

### The Association Between MD2 Expression and Clinicopathologic Features

**Figure 2:**
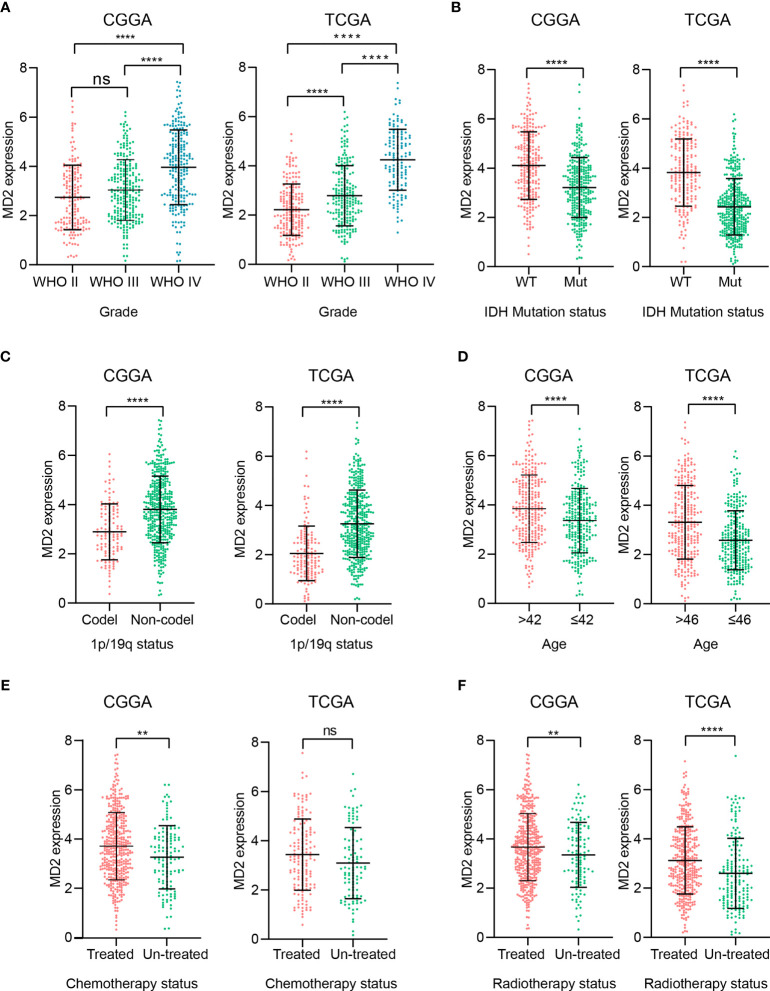
The association between MD2 expression and clinicopathologic characteristics. **(A)** The correlation analysis between MD2 expression level and WHO grade in CGGA and TCGA datasets. **(B)** The relationship analysis between MD2 expression level and IDH1 status in CGGA and TCGA datasets. **(C)** The association analysis between MD2 expression level and 1p/19q status in CGGA and TCGA datasets. **(D)** The correlation analysis between MD2 expression level and glioma patients’ age in CGGA and TCGA datasets. **(E)** The association analysis between MD2 expression level and chemotherapy in CGGA and TCGA datasets. **(F)** The relationship analysis between MD2 expression level and radiotherapy in CGGA and TCGA datasets. WT, Wildtype; Mut, Mutant. ** and **** indicate *p* < 0.01 and *p* < 0.0001, respectively; ns, not statistically significant.

To elucidate potential roles of MD2 in the malignant progression of gliomas, we analyzed its expression levels in different grades of glioma in the datasets of CGGA and TCGA. Although there was no significant difference of MD2 expression between grade II and III in the dataset of CGGA, MD2 expression level was significantly increased along with the progression of gliomas from grade II to grade IV in both datasets (**Figure 2A**). Since IDH1 mutation is recognized as a principal driver in low grade gliomas, with an incidence of more than 70% (45, 46), we therefore examined the relationship between MD2 expression and the status of IDH1. In both databases from CGGA and TCGA, patients with higher MD2 expression level were synchronized with wild-type IDH1, whereas most of those with lower MD2 expression was associated with IDH1 mutation (**Figure 2B**). In parallel, 1p/19q codeletion is an important clinicopathologic characteristic for gliomas progression, and codeleted patients usually survive longer than non-codeleted patients (46, 47). Thus, we assessed the potential clinical association between MD2 expression and the status of 1p/19q in both databases of CGGA and TCGA. The analyses indicated that MD2 expression was significantly upregulated in 1p/19q non-codeleted group compared to the patients with 1p/19q codeleted (**Figure 2C**). We further analyzed the expression of MD2 in glioma patients with different ages in the databases of CGGA and TCGA. According to the median ages of patients, the glioma patients were separated into high-age (> 43 or > 46 years) and low-age (≤ 42 or ≤ 46 years) groups. The results indicated that the expression level of MD2 was significantly lower in the low-age group than that in the high-age group (**Figure 2D**). In addition, we found that the expression level of MD2 was increased after chemotherapy and radiotherapy, suggesting that the expression of MD2 may be related to therapeutic resistance (**Figures 2E, F**). These results indicated that high expression of MD2 was correlated with faster progression of gliomas.

### The Association Between *MD2* Gene Methylation and Clinicopathologic Features

**Figure 3:**
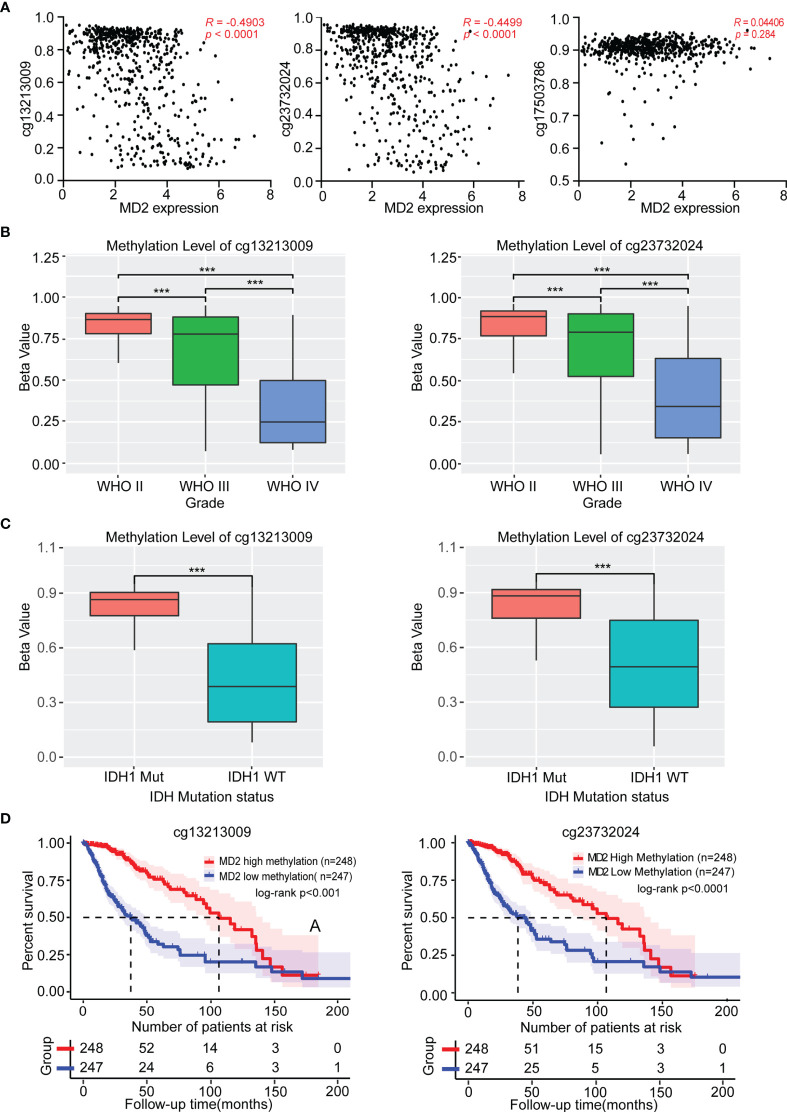
The association between MD2 methylation and clinicopathologic characteristics. **(A)** The correlation between MD2 expression level and its DNA methylation at cg13213009, cg23732024 and cg17503786 CpG sites. **(B)** The relationship of MD2 DNA methylation level at cg13213009 or cg23732024 CpG sites with WHO grade. **(C)** The association of MD2 DNA methylation level at cg13213009 or cg23732024 CpG sites with IDH status. **(D)** Kaplan-Meier curves of low and high MD2 DNA methylation at cg13213009 or cg23732024 CpG sites. WT, Wildtype; Mut, Mutant. *** indicates *p* < 0.001.

To investigate the causes of abnormal expression of MD2 in gliomas, we detected MD2 expression level and its DNA methylation status. As shown in **Figure 3A**, we observed an obviously negative correlation between MD2 expression and its DNA methylation at two CpG sites including cg13213009 (*R* = -0.4903, *p* < 0.0001) and cg23732024 (*R* = -0.4499, *p* < 0.0001), while the methylation at cg17503786 did no correlate with MD2 expression. Subsequently, we selected the sites of cg13213009 and cg23732024 CpG to further examine the prognostic values of MD2 methylation in glioma patients. Consequently, MD2 methylation levels at both CpG sites significantly decreased in accordance with the progression of gliomas from grade II to grade IV (**Figure 3B**). Next, we established the relationship between MD2 methylation level and the status of IDH1. The results showed that higher methylation levels at both CpG sites tend to be associated with IDH1 mutation (**Figure 3C**). Moreover, Kaplan-Meier plots indicated that lower DNA methylation of *MD2* gene correlates with shorter overall survival of glioma patients (*p* < 0.001) based on the analyses of CGGA and TCGA databases (**Figure 3D**). Collectively, these analyses demonstrated that *MD2* gene methylation was also associated with the progression of glioma and the increased expression level of MD2 in glioma was induced by its reduction of DNA methylation.

### The Roles of MD2 as an Independent Risk Factor

**Figure 4:**
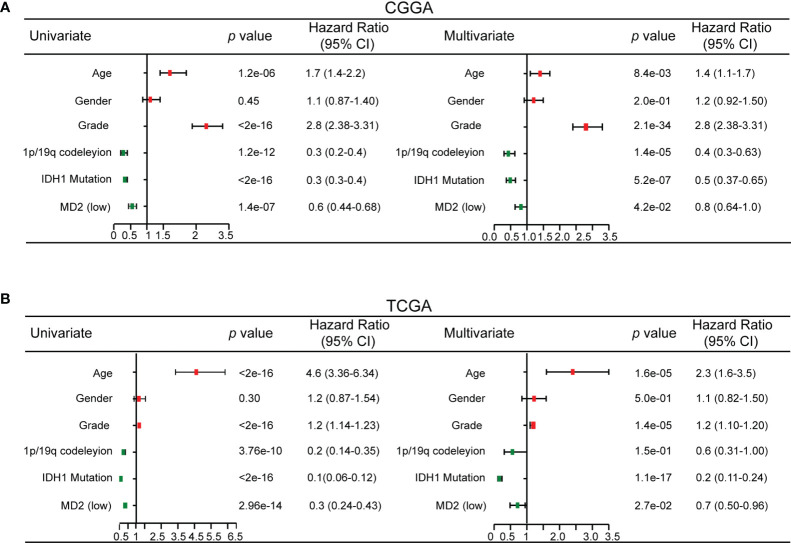
Univariate and multivariate Cox regression analysis. **(A)** Univariate and multivariate Cox regression analyses risk score of MD2 expression level and several related clinical variables in CGGA database. Red color indicates disadvantageous factors, HR>1, Green color indicates protective factors, HR < 1. **(B)** Univariate and multivariate Cox regression analyses risk score of MD2 expression level and several related clinical variables in TCGA database. Red color indicates disadvantageous factors, HR>1, green color indicates protective factors, HR<1.

**Figure 5:**
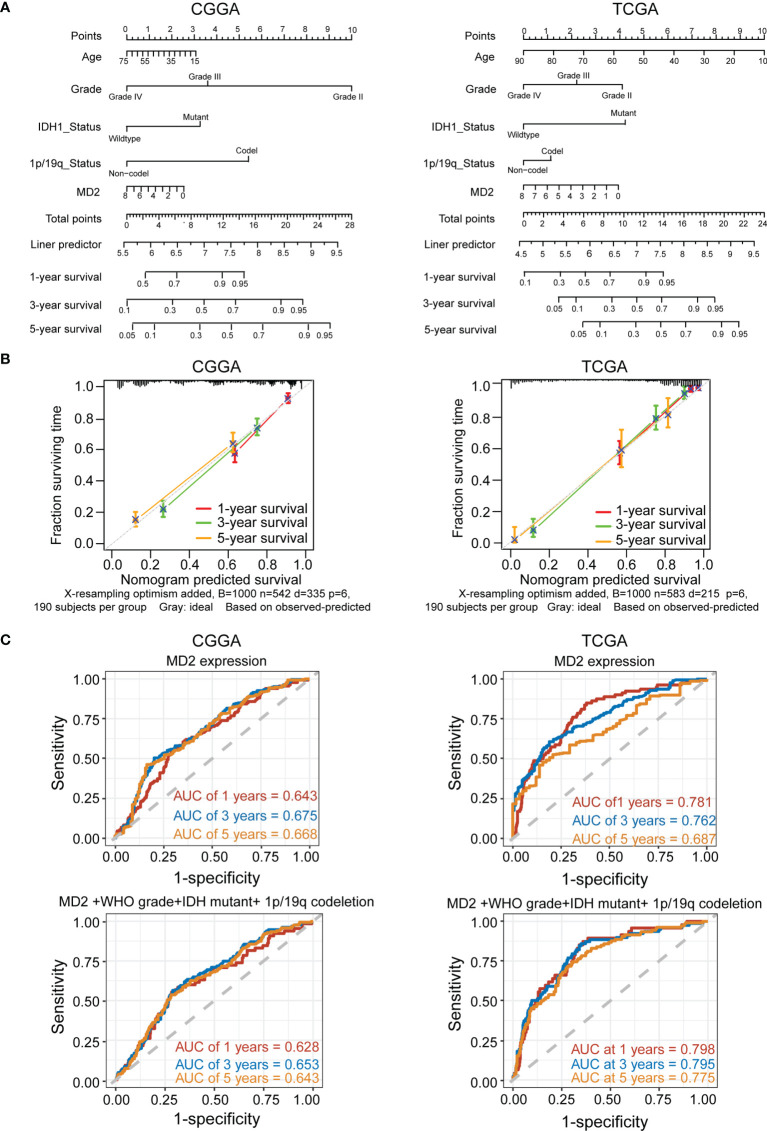
MD2 acted as an independent risk factor of poor prognosis in glioma patients. **(A)** The nomogram was developed by integrating the MD2 expression with clinicopathologic features, age, grade, IDH status and 1p/19q status in the CGGA (left) and TCGA (right) databases. **(B)** The calibration plot of the nomogram for predicting overall survival at 1-year (red), 3-year (green) and 5-year (orange), ideal line as control (grey) in the CGGA (left) and TCGA (right) databases. **(C)** ROC curves analysis for the 1-. 3-, and 5-year survival base on the CGGA (left) and TCGA (right) databases.

To explore whether MD2 is an independent and significant factor for the prognosis of gliomas, we carried out univariate and multivariate Cox regression analyses using the data from CGGA and TCGA. The results indicated that MD2 expression (univariate hazard ratio (HR): 0.6, *p* = 1.4e-07; multivariate HR: 0.8, *p* = 4.2e-02), 1p/19q codeletion (univariate HR:0.3, *p* = 1.2e-12; multivariate HR: 0.4, *p* = 1.4e-05) and IDH1 mutation (univariate HR:0.3, *p* < 2e-16; multivariate HR: 0.5, *p* = 5.2e-07) could serve as independent protectable variances for gliomas, and WHO grade (univariate HR:2.8, *p* < 2.6e-16; multivariate HR: 2.8, *p* = 2.1e-34) and age (univariate HR:1.7, *p* = 1.2e-06; multivariate HR: 1.4, *p* = 8.4e-03) could be risk factors (**Figure 4A**). Similar results were also obtained by using the data from TCGA (**Figure 4B**). Next, we constructed the nomograms with these independent prognosis factors (Age, WHO grade, IDH1 status, 1p/19q status and MD2 expression) to predict 1-, 3- and 5-year survival probability of each glioma patient (**Figure 5A**). The calibration plot for the probability of survival revelated that the nomogram-predicated survival probability was very close to the ideal reference line in the databases of CGGA and TCGA, and the C-index were 0.75 in CGGA dataset and 0.86 in TCGA dataset, respectively (**Figure 5B**). In addition, the areas under ROC curve (AUC) of MD2 expression is 0.643 in 1-year survival, 0.675 in 3-year survival and 0.668 in 5-year survival in CGGA database and is 0.781 in 1-year survival, 0.762 in 3-year survival and 0.687 in 5-year survival in TCGA database (**Figure 5C**). Since WHO grade, IDH mutant status and 1p/19q codeletion are important clinicopathologic characteristics for glioma progression, we further performed ROC analysis combining MD2 expression with these parameters. As a result, the AUC of 1-, 3-, and 5-year survival rates in CGGA database were 0.643, 0.675 and 0.668, respectively. Similar results were also obtained in TCGA database (**Figure 5C**). These results suggested that MD2 is an independent factor for the prognosis of glioma.

### The Predicted Functions of MD2 in Gliomas

**Figure 6:**
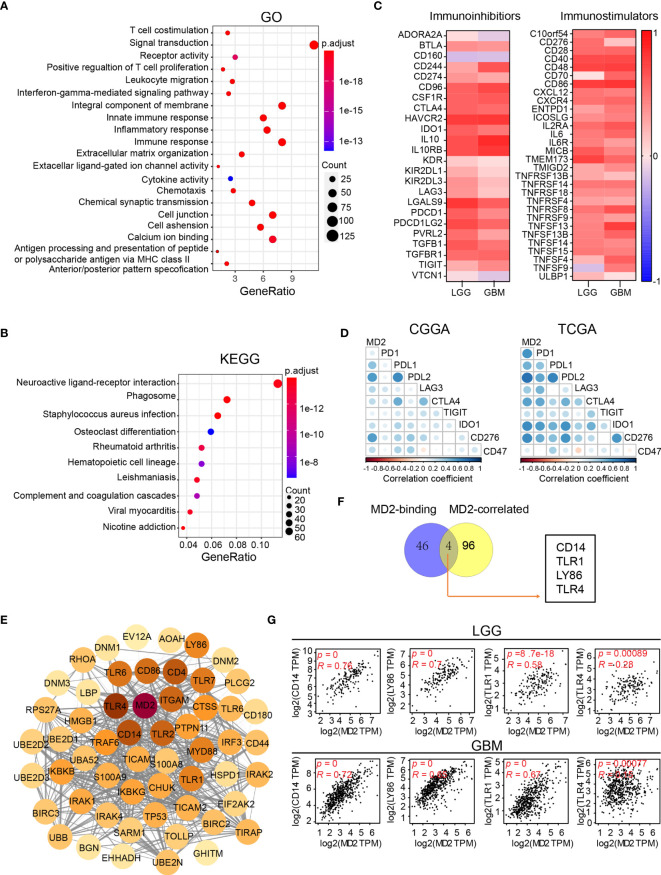
Functional enrichment analysis of MD2. **(A)** Bubble plot of GO enrichment analysis of MD2-related signal pathway. **(B)** Bubble plot of KEGG enrichment analysis of MD2-related signal pathway. Node size indicates the number of contained in the corresponding GO/KEGG term, and the color of the node indicates the *p*-value. **(C)** The heatmap of correlation between the immunoinhibitors and MD2 expression in LGG and GBM (left); the heatmap of correlation between the immunostimulators and MD2 expression in LGG and GBM (right). **(D)** Correlation analysis of MD2 expression and nine immune-related checkpoints. **(E)** Protein-protein interaction network based on MD2-binding proteins and top 100 genes correlated with MD2 expression. **(F)** Interactive Venn diagram to analyze the overlapping genes between MD2-binding proteins and top 100 genes correlated with MD2 expression. **(G)** The correlation between the expression of MD2 and CD14, LY86, TRL1 and TRL4 in LGG and GBM.

To dissect the biological functions of MD2 in gliomas, we performed GO and KEGG enrichment analyses based on MD2 expression. The GO functional analyses indicated that a variety of functions are associated with MD2, among which the major functions are linked to T-cell costimulation, innate immune responses and inflammatory responses (**Figure 6A**). Meanwhile, KEGG pathway analyses showed that MD2-related pathways involve neuroactive ligand-receptor interaction, phagosome, infection and leishmaniasis (**Figure 6B**). To clarify these results, we firstly examined MD2-related immune-inhibitors and immune-stimulators using TISIDB database. We found that 8 immunoinhibitors (CD96, CSF1R, HAVCR2, IL10, IL10RB, LGALS9, PDCD1LG2, and TGFBR1, *R* > 0.5) and 6 immunostimulators (CD28, CD40, CD48, CD86, IL2RA, TMEM173, *R* > 0.5) were significantly associated with MD2 in gliomas (**Figure 6C**). Then, we detected the association between MD2 expression and nine immune checkpoints (PD1, PDL1, PDL2, LAG3, CTLA4, TIGIT, IDO1, CD276, CD47), which are promising immunotherapeutic targets for gliomas (48, 49). The analysis revealed that MD2 is positively associated with PDL1, PDL2 and CD276 (**Figure 6D**). To further analyze the potential roles of MD2 in glioma progression, MD2-binding proteins and genes correlated with MD2 expression were identified using the databases of STRING and GEPIA2. Fifty MD2-binding proteins and top 100 genes correlated with MD2 were obtained and the PPI network of those genes was mapped as shown in **Figure 6E**. The Venn diagram indicated that four common members, including CD14, LY86, TLR1 and TLR4, occur in both groups (**Figure 6F**), suggesting their regulatory roles in mediating the innate immune responses and inflammatory responses. The subsequent correlation analyses also indicated MD2 was strongly correlated with CD14, LY86, TLR1 and TLR4 in gliomas (**Figure 6G**). Through these functional and pathway enrichments, we found that MD2 strongly correlates with immunological responses in gliomas.

### The Correlation Between MD2 and Immune Cell Infiltration in Gliomas

**Figure 7:**
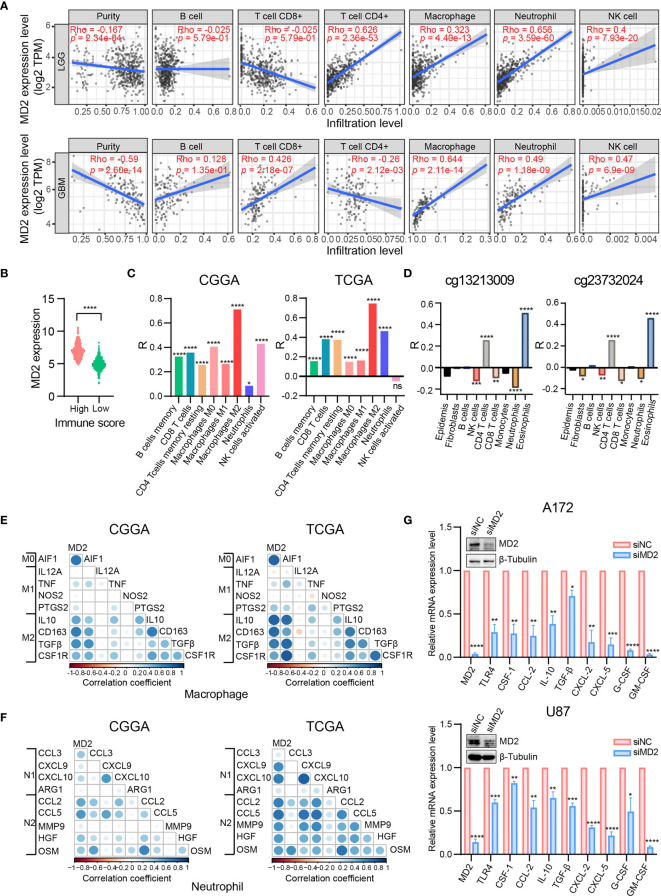
Correlation analysis between MD2 expression and immune cells filtration. **(A)** The correlation of MD2 with the six types of immune cells filtration level in LGG and GBM based on the TIMER algorithm. **(B)** Comparison of MD2 expression between the high and low immune score groups of gliomas. **(C)** The correlation between MD2 and eight types of immune cells and subpopulations filtration level in CGGA and TCGA datasets based on CIBERSORT algorithm. **(D)** The correlation between the MD2 DNA methylation and nine types of immune cells filtration levels. **(E)** The correlation between MD2 and classical phenotype markers of M0, M1 and M2 in CGGA and TCGA databases. Color depth and circle square represent the degrees of correlation. **(F)** The correlation between MD2 and classical phenotype markers of N1 and N2 in CGGA and TCGA databases. Color depth and circle square represent the degrees of correlation. **(G)** qRT-PCR analysis of the cytokine mRNA expression after MD2 knockdown in U87 and A172 cells. *, **, *** and **** indicate *p* < 0.05, *p* < 0.01, *p* < 0.001 and *p* < 0.0001, respectively.

Given the involvement of MD2 in immunomodulatory signaling pathways in gliomas, we explored the association between MD2 expression and immune cell infiltration. The TIMER algorithm was used to examine the correlation between MD2 expression and six types of immune cell infiltration in LGG and GBM, respectively. MD2 showed a significantly positive correlation with the infiltrations of macrophages, neutrophils and NK cells in both LGG and GBM (**Figure 7A**). Meanwhile, the immune scores were calculated by ESTIMATE database, showing that immune scores were positively related with MD2 expression (*p* < 0.0001) (**Figure 7B**). Additionally, the correlation between MD2 and 22 types of infiltrating immune cells was calculated by CIBERSORT algorithm using the data from CGGA and TCGA databases. Different from the analysis results by TIMER, in addition to macrophages and neutrophils, three other immune infiltrating populations, including B cells, CD4^+^ T cells and CD8^+^ T cells, were also significantly and positively correlated with MD2 expression (**Figure 7C** and **Table 2**). Furthermore, we found that DNA methylation of *MD2* gene was negatively correlated with the infiltration of neutrophils, which is consistent with our analysis (**Figure 7D**). The correlation between MD2 expression and classical phenotypes of macrophages and neutrophils were analyzed in the databases of CGGA and TCGA, and we discovered that MD2 possesses an exceptionally positive correlation with M0 and M2 markers of TAMs (M2-type macrophages promote tumor progression) instead of M1 marker (M1-type macrophages inhibit tumor progression) (**Figure 7E**). Similarly, MD2 also showed a positive correlation with N2 phenotype marker of TANs (**Figure 7F**). The correlations between MD2 expression and the expression of other immune cell-specific markers were further confirmed by analyzing the database of TIMER as shown in **Table 3**.

**Table 2:** Correlation between MD2 and immune cell subtype in glioma.

Immune cell subtype	CGGA database		TCGA database	
	*R*	*p* value	*R*	*p* value
B cells naive	0.036076	0.342977	-0.12421	**0.002403**
B cells memory	0.32466	**0**	0.155444	**0.000141**
Plasma cells	0.244242	**7.17E-11**	-0.31393	**4.44E-15**
T cells CD8	0.357901	**0**	0.380746	**0**
T cells CD4 naive	-0.08797	0.020555	-0.24484	**1.43E-09**
T cells CD4 memory resting	0.256971	**6.50E-12**	0.372284	**0**
T cells CD4 memory activated	0.214495	**1.18E-08**	0.366744	**0**
T cells follicular helper	0.283902	**2.58E-14**	-0.029	0.480172
T cells regulatory Tregs.	0.209735	2**.50E-08**	0.283553	**1.83E-12**
T cells gamma delta	0.315129	**0**	-0.03139	0.444718
NK cells resting	0.048392	0.203244	0.413341	**0**
NK cells activated	0.430056	**0**	-0.04948	0.228169
Monocytes	0.41221	**0**	0.260145	**1.17E-10**
Macrophages M0	0.40638	**0**	0.149611	**0.00025**
Macrophages M1	0.264746	**1.40E-1**2	0.161756	**7.39E-05**
Macrophages M2	0.711729	**0**	0.745743	**0**
Dendritic cells resting	0.06904	**0.069317**	0.089228	**0.029536**
Dendritic cells activated	0.052962	0.163716	0.08712	**0.033616**
Mast cells resting	0.212427	**1.64E-08**	0.155258	**0.000143**
Mast cells activated	0.255813	**8.13E-12**	-0.03399	0.407893
Eosinophils	0.021589	0.570466	0.035063	0.393248
Neutrophils	0.085387	**0.024585**	0.463626	**0**

The bold indicates statistical significance.

**Table 3:** Correlation between MD2 and immune cell-specific markers in glioma.

Immune cell types	Markers	LGG		GBM	
		*R*	*p* value	*R*	*p* value
CD8+ T cell	CD8A	0.227	**1.96e-07**	0.388	**7.36e-07**
	CD8B	0.31	**6.29e-13**	0.43	**2.92e-09**
T cell	CD2	0.65	**2.66e-26**	0.607	**8.79e-17**
	CD3D	0.592	**4.84e-50**	0.65	**1e-19**
	CD3E	0.621	**2.02e-56**	0.591	**9.41e-16**
B cell	CD27	0.441	**5.17e-26**	0.149	0.066
	CD19	0.243	**2.18e-08**	0.534	**1.18e-12**
	CD79A	0.384	**1.42e-19**	0.256	**1.41e-03**
Monocyte	CD14	0.757	**5.63e-97**	0.705	**2.41e-24**
	CD86	0.733	**5.1e-88**	0.691	*4.52e-23*
TAM	CCL2	0.539	**3.84e-40**	0.607	**9e-17**
	CD68	0.816	**3.33e-12**4	0.649	**1.24e-19**
	IL10	0.644	**7.22e-62**	0.756	**1.33e-29**
Neutrophil	CCR7	0.439	**1.1e-25**	0.484	*2.37e-10*
	ITGAM	0.625	**2.86e-57**	0.491	**1.19e-10**
Natural killer cell	KIR2DL1	0.031	0.485	0.157	0.053
	KIR2DL2	0.194	**9.27e-04**	0.12	0.138
	KIR2DL3	0.251	**7.1e-09**	0.028	0.731
Dendritic cell	CD1C	0.479	**6.01e-37**	0.434	**2.11e-08**
	CD209	0.342	**1.34e-15**	0.255	**1.48e-03**
	NRP1	0.347	**5.29e-16**	0.333	**2.55e-05**
Th1 cell	ITGAX	0.533	**3.31e-39**	0.268	**8.13e-04**
	STAT1	0.439	**1.01e-25**	-0.021	0.798
	STAT4	-0.206	**2.46e-06**	0.284	**3.71e-04**
Th2 cell	GATA3	0.441	**6.13e-26**	0.167	0.039
	STAT6	0.371	**2.71e-18**	0.43	*2.86e-08*
Tfh cell	BCL6	0.055	0.21	-0.139	0.085
	IL21	0.096	0.029	0.005	0.95
Th17 cell	IL17A	0.009	0.83	-0.049	0.546
	STAT3	0.513	**6.01e-36**	-0.109	0.179
T cell exhaustion	CTLA4	0.46	**2.53e-28**	0.468	**1.08e-09**
	LAG3	0.334	**6.11e-15**	0.023	0.778
	HAVCR2	0.746	**7.28e-93**	0.684	**1.77e-22**
	PDCD1	0.571	**5.2e-46**	0.295	**2.11e-04**
Treg cell	CCR8	0.232	**9.91e-8**	0.4	**2.93e-07**
	FOXP3	-0.151	**5.61-04**	0.166	**4.09e-02**

The bold indicates statistical significance.

To ensure the correlation between MD2 expression and the infiltrations of macrophages and neutrophils, MD2 was knocked-down using siRNA in U87 and A172 glioma cells, resulting in remarkable decrease of the expression of MD2 at mRNA and protein levels (**Figure 7G**). Subsequently, we detected the changes of mRNA expression of the cytokines secreted by glioma cells. Both M2-type TAMs-related polarization factors (CSF-1, CCL-2, IL-10 and TGF-β) and N2-type TANs-related polarization factors (CXCL-2, CXCL-5, G-CSF and GM-CSF) were markedly decreased after the knockdown of MD2 in A172 and U87 cells (**Figure 7G**). At the same time, mRNA level of TLR4 was also dramatically reduced by silencing MD2 in A172 and U87 cells (**Figure 7G**). Collectively, these data revealed that MD2 expression is significantly associated with macrophage and neutrophil infiltration to promote their polarization toward M2-TAMs or N2-type TANs.

## Discussion

Gliomas are the most common primary brain tumors and possess high heterogeneity and invasiveness. Conventional chemotherapeutic strategies are unable to completely eliminate residual tumor tissue and further generate inevitable recurrence and drug-resistance (50, 51). To overcome the limitations of current standard therapy for gliomas, newer therapeutic strategies have been developed over the past decades. Immunotherapy has emerged as an alternative therapy to deal with the intolerance or relapse of glioma to traditional therapies (11, 12). Although it has been well-known that tumor-infiltrating immune cells (TIICs) in the tumor microenvironment could regulate the invasion and immune evasion of tumor cells and affect the therapeutic effect of cancer (20, 52, 53), there is still a lack of reliable biomarkers for early diagnosis and the prediction of therapeutic effectiveness. Consequently, the therapeutic outcomes and overall survivals for glioma patients remain unsatisfactory, and the discovery of novel biomarkers could monitor the situation of immune cell infiltration for the guidance of immunotherapy.

In the present study, we found that MD2 was significantly upregulated in gliomas and its mRNA expression was negatively regulated by its DNA methylation. Further functional studies unveiled that MD2 was closely related to the infiltrations of TAMs and TANs. In addition, we further confirmed that MD2 was able to promote the polarization of TAMs and TANs to their immunosuppressive subtypes (M2 or N2) by elevating the secretion of related cytokines in glioma cancer cells.

With the compelling evidence on the association between inflammation and cancer (31, 54), the involvement of TLR4 in tumor progression has been well recognized, such as in breast cancer (55), colorectal cancer (33) and ovarian cancer (56). By contrast, the roles of the coreceptor of TLR4, MD2, in tumor progression remain poorly understood. Although MD2 has been reported to be overexpressed in breast cancer and colon cancer cells to promote proliferation, migration and invasion of tumor cells in recent years (57–59), the expression of MD2, its association with tumor progression and its functional roles in gliomas are unclear. In the present study, MD2 was proved to serve as an independent prognostic factor to predict overall survivals of glioma patients. Although WHO grade, IDH status and 1p/19q status are the most commonly used clinical prognostic parameters, several new prognostic indicators for gliomas have been identified in the past few years (60, 61). For example, upregulation of Piezo1 was reported as an independent prognostic factor to adversely affect the prognosis of patients (62). Similar to other parameters, MD2 expression was positively associated with these clinicopathologic characteristics in gliomas, which provides additional biomarker for more accurate prognosis prediction. Different from the other new prognostic models, the mechanism of action for our model was preliminarily established. ROC analysis further verified that MD2 could serve as a sensitive indicator to predict the 1-year, 3-year, and 5-year survival rates of the patients, indicating the value of MD2 as a prognostic biomarker for gliomas.

Abnormal DNA methylation plays an important role in various types of tumorigenesis (63, 64). In gliomas, the methylation status of the promoter for O6-methylguanine-DNA methyltransferase has been identified to associate with the progression of disease and to correlate with the sensitivity of glioma patients for TMZ treatment (65, 66). In addition, other genes have also been reported as DNA-methylation-based biomarkers of gliomas, such as CXCR4, ST6Gal1, SFRP1 (67–69). In the present study, we found that mRNA expression level of MD2 was negatively correlated with its DNA methylation, which may explain, at least in part, the high expression of MD2 in glioma tissues. Additional analysis also showed that lower DNA methylation level of MD2 correlates with worse overall survival and more malignant clinicopathological phenotypes. Taken together, MD2 can be used as an independent prognostic biomarker for gliomas.

The tumor microenvironment is a complex system consisting of tumor cells, infiltrating lymphocytes, immune cells, fibroblasts and endothelial cells, which is closely related to tumor initiation, malignant progression and metastasis (20, 52). Macrophages and neutrophils are the main components of tumor infiltrating cells (70). In the tumor microenvironment, macrophages could be polarized to either anti-tumor (M1) or pro-tumor (M2) phenotype in response to different stimuli (71). Similarly, neutrophils can also polarize to anti-tumor (N1) or pro-tumor (N2) phenotypes in the tumor microenvironment (72). Due to the fact that the extent of macrophages and neutrophils infiltration is significantly correlated with the grade and clinical prognosis of gliomas (21, 73, 74), we were quite interested in clarifying the relationship of MD2 expression and immune cell infiltration. When KEGG and GO enrichment analysis was performed, the major biological functions of MD2 were clustered to immune-related pathways. Given that several molecules positively correlated with immune cell infiltration, such as, APOBEC3B and TNFSF13, have been reported as potential biomarkers in gliomas (75, 76), to ensure our findings, we utilized the TIMER, ESTIMATE and CIBERSORT algorithms to further assess the association between MD2 expression and immune cells infiltration. The strong correlation between MD2 expression and macrophage and neutrophil infiltration implied that MD2 not only was a prognostic biomarker, but also a potential player in the tumor microenvironment. Furthermore, the positive correlation between MD2 expression and specific markers of M2-type TAMs and N2-type TANs provided additional evidence to support the involvement of MD2 in the microenvironment of gliomas. Moreover, in glioma U87 and A172 cells, the reduction of the expression of related cytokines secreted by glioma cells from the silencing of MD2 strongly confirmed the capability of MD2 on the promotion of polarizing to M2 and N2 phenotypes with respect to macrophages and neutrophils, in which detailed molecular mechanisms and events require future investigations.

The discovery of the correlation between mRNA level of MD2 and the resistance to chemotherapy and radiotherapy may promote further investigations on the mechanism of drug-resistance in glioma patients, which could better direct clinical practice. Meanwhile, based on comprehensive nomogram predictive model analysis, MD2 exhibits great potential in clinical application. Given that glioma patients are usually immunosuppressed, and the infiltration and polarization of macrophage and neutrophils are the major cause for glioma resistance to chemotherapy and radiotherapy (21, 77), it was reasonable to predict that MD2 may be involved in the process of drug resistance by regulating immune response in glioma. To evaluate the potential of MD2 as a potential treatment target, the prediction of drugs that may target or bind MD2 was conducted using BindingDB database (78). Although 34 small molecules were identified to be able to interact with MD2 (data not shown), none of them has been reported in the treatment of glioma. Therefore, the therapeutic utility of these small molecules requires further investigation.

In summary, we have revealed that MD2 was upregulated in gliomas patients, and the expression of MD2 was negatively regulated by the level of DNA methylation. The strong correlation between MD2 expression and glioma progression and the close association with immune cell infiltration have warranted that MD2 can be used as a novel biomarker for clinical prognosis of gliomas.

## Data Availability Statement

The datasets analyzed for this study can be found in the CGGA (www.cgga.org.cn), TCGA (http://xena.ucsc.edu/) and Rembrandt (https://wiki.cancerimagingarchive.net/display/Public/REMBRANDT).

## Author Contributions

MZ: Investigation, Data curation and Writing —Original draft preparation. XL: Investigation and Validation. YC: Writing —Review and Editing. SW: Supervision, Writing —Review and Editing. All authors contributed to the article and approved the submitted version.

## Funding

This work was supported by grants from “Double First-Class” University Project (CPU2018GY36), and the Priority Academic Program Development of Jiangsu Higher Education Institutions (PAPD).

## Conflict of Interest

The authors declare that the research was conducted in the absence of any commercial or financial relationships that could be construed as a potential conflict of interest.

## Publisher’s Note

All claims expressed in this article are solely those of the authors and do not necessarily represent those of their affiliated organizations, or those of the publisher, the editors and the reviewers. Any product that may be evaluated in this article, or claim that may be made by its manufacturer, is not guaranteed or endorsed by the publisher.
