# Supplementary figures and images for: MD2 Is a Potential Biomarker Associated with Immune Cell Infiltration in Gliomas
Source: Front Oncol. 2022 Mar 17;12:854598. doi: 10.3389/fonc.2022.854598 (PMC8968038; doi:10.3389/fonc.2022.854598)

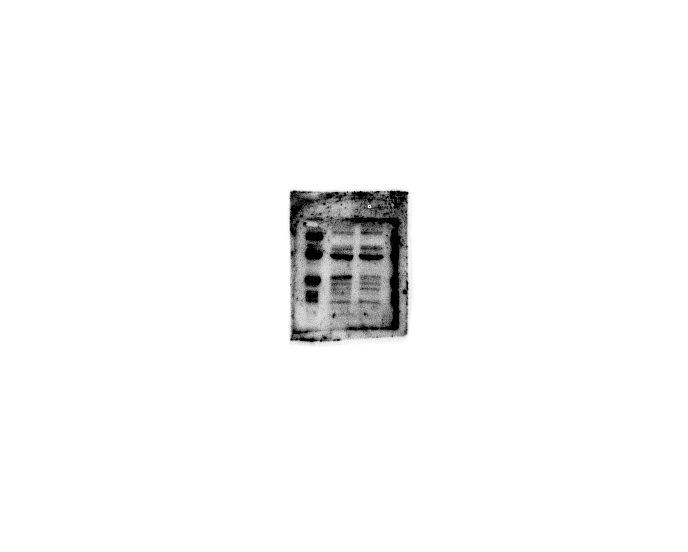

Supplement: Supplementary file 1 [file DataSheet_1.zip › Original gels/Fig. 6F A172 cell-MD2.tif]

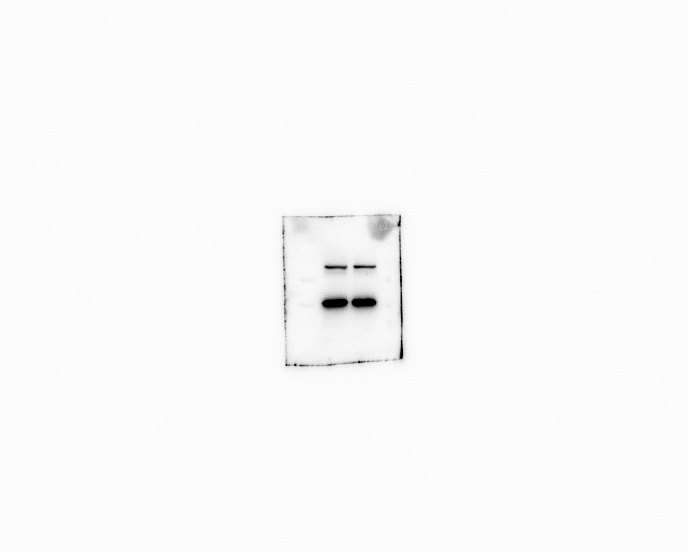

Supplement: Supplementary file 1 [file DataSheet_1.zip › Original gels/Fig. 6F A172 cell-β-Tubulin.tif]

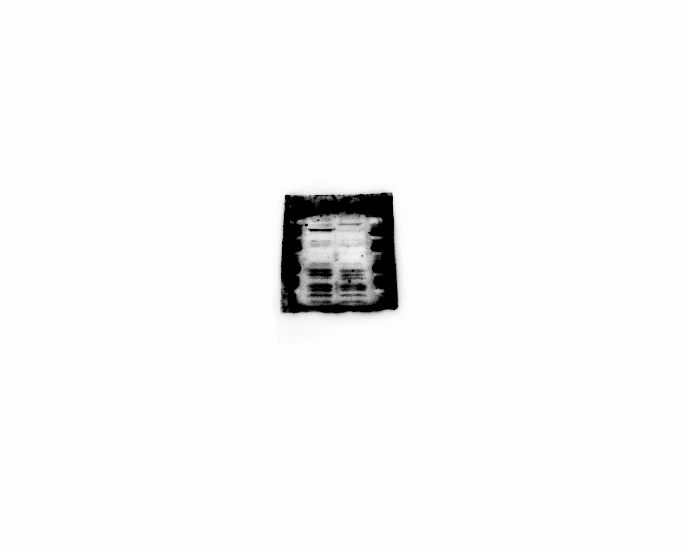

Supplement: Supplementary file 1 [file DataSheet_1.zip › Original gels/Fig. 6F U87 cell-MD2.tif]

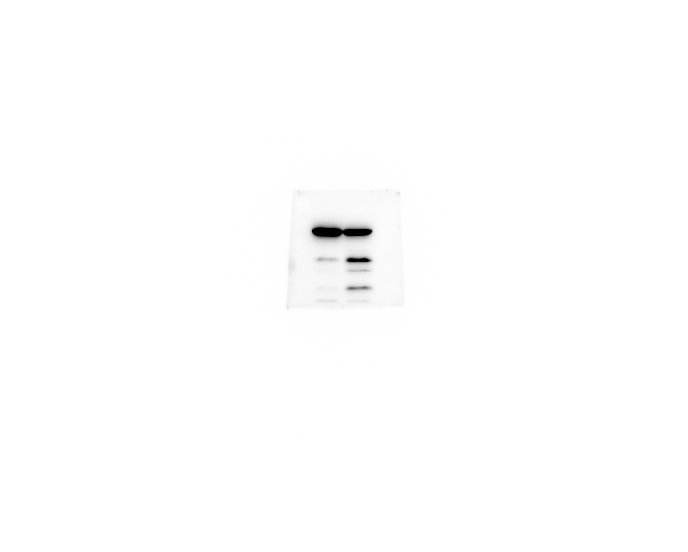

Supplement: Supplementary file 1 [file DataSheet_1.zip › Original gels/Fig. 6F U87 cell-β-Tubulin.tif]
