# Supplementary material for: MD2 Is a Potential Biomarker Associated with Immune Cell Infiltration in Gliomas
Source: Front Oncol. 2022 Mar 17;12:854598. doi: 10.3389/fonc.2022.854598 (PMC8968038; doi:10.3389/fonc.2022.854598)
Supplement: Supplementary file 2 [file DataSheet_2.zip › Source data/Data download websites.docx]

**Source data for Figure 1A**

TcgaTargetGtex_rsem_gene_fpkm.gz

<https://toil-xena-hub.s3.us-east-1.amazonaws.com/download/TcgaTargetGtex_rsem_gene_fpkm.gz>

**Source data for Figure 1E, Figure 2, Figure 5A and B**

TCGA_survival_data

<https://toil-xena-hub.s3.us-east-1.amazonaws.com/download/TCGA_survival_data>;

TcgaTargetGTEX_phenotype.txt.gz

<https://toil-xena-hub.s3.us-east-1.amazonaws.com/download/TcgaTargetGTEX_phenotype.txt.gz>;

TCGA.GBMLGG.sampleMap-GBMLGG_clinicalMatrix

<https://tcga-xena-hub.s3.us-east-1.amazonaws.com/download/TCGA.GBMLGG.sampleMap%2FGBMLGG_clinicalMatrix>;

**Source data for Figure 3 and Figure 5C**

HumanMethylation450

<https://tcga-xena-hub.s3.us-east-1.amazonaws.com/download/TCGA.GBMLGG.sampleMap%2FHumanMethylation450.gz>;

methylation450 probeMap derived from GEO GPL13534

**Source data for Figure 1A, 1D and Figure 2**

CGGA.mRNAseq_693.RSEM-genes.20200506.txt

CGGA_RNAseq_Control_20.txt

CGGA.mRNAseq_693_clinical.20200506.txt

<http://www.cgga.org.cn/download.jsp>
